# Incorporating graphene-modified mica and conductive nickel particles for enhanced corrosion resistance in epoxy zinc-rich coatings

**DOI:** 10.3389/fchem.2025.1544762

**Published:** 2025-04-30

**Authors:** Yong Jiang, Haiping Zhang, Hui Zhang, Yuanyuan Shao, Jesse Zhu, Xuliang Jin

**Affiliations:** ^1^ School of Chemical Engineering and Technology, Tianjin University, Tianjin, China; ^2^ School of Chemical Engineering and Light Industry, Guangdong University of Technology, Guangzhou, China; ^3^ Nottingham Ningbo China Beacons of Excellence Research and Innovation Institute, The University of Nottingham Ningbo China, Ningbo, China; ^4^ Department of Chemical and Biochemical Engineering, Western University, London, ON, Canada; ^5^ Datang North China Electric Power Test and Research Institute, Beijing, China

**Keywords:** epoxy zinc-rich coatings, anti-corrosion coatings, conductive nickel, graphene, conductive mica

## Abstract

Epoxy zinc-rich coatings usually require high zinc content to ensure its anti-corrosion performance. However, excessive zinc powder content will reduce the mechanical properties of the coating, increase the economic cost, harm the environment, etc. Therefore, this paper aims to reduce the amount of zinc powder and improve the corrosion performance of epoxy zinc-rich coatings by introducing two kinds of conductive particle materials, conductive graphene-mica powder and conductive nickel. Conductive graphene was first loaded on mica powder and the obtained conductive graphene-mica powder and the conductive nickel were introduced to the epoxy zinc-rich coatings to partially replace zinc component. The anti-corrosion properties of the coating were systematically evaluated by EIS and salt spray test. The resulting epoxy zinc-rich coating with nickel powder or conductive graphene-mica demonstrates outstanding salt spray resistance, lasting up to 2,000 h, exhibiting superior anti-corrosion performance at reduced zinc content of 60% or 45% compared to conventional coatings with 70% pure zinc powder. This study introduces a novel conductive mica material and investigates conductive metal nickel additive, effectively reducing zinc content in epoxy zinc-rich coatings, which offers valuable insights for developing high-performance anti-corrosion coatings.

## 1 Introduction

Epoxy zinc-rich coatings are widely used in the protection of steel materials due to their excellent physical, mechanical and anti-corrosion properties. To achieve its anti-corrosion effect, the zinc content of epoxy zinc-rich coatings is generally above 70 wt.% to ensure continuous electrical connection and a reliable penetration path in the coating. However, a high zinc powder content will introduce some negative effects, such as low adhesion strength, weak impact resistance, high economic costs, and environmental concerns. For example, during the hot working and production of zinc coated components, a large amount of zinc oxide is released, potentially leading to “zinc fever” among workers. To mitigate the negative impact of high zinc powder content, numerous researchers have suggested partially substituting zinc powder with conductive additives (Hayatdavoudi and Rahsepar, 2017; Park and Shon, 2015; Ramezanzadeh et al., 2017).

Some studies have found that conductive nanomaterials such as graphene (He et al., 2024; Lan et al., 2023; Wang et al., 2020; Zhang and Zheng, 2023), carbon nanotube (Son et al., 2024; Gergely et al., 2014), graphene derivative (Fang et al., 2024; Zhou et al., 2019) and nanocrystalline diamonds (Kratochvílová et al., 2019) can partially replace zinc powder in epoxy zinc-rich coatings without compromising their anti-corrosion performance. For example, He et al. (2024) clarified the essence of graphene enhancing the protective performance of zinc-rich coatings. The introduced graphene tended to promote the corrosion of zinc particles, building chemical shielding effects by converting H_2_O and O_2_, etc. and then enhancing the physical shielding performance through the spontaneous formation of corrosion products. Zhang and Zheng (2023) prepared a series of graphene-modified zinc-rich epoxy coatings. The contact mode of zinc particles-graphene-zinc particles can be formed between graphene and zinc particles in coatings, thus improving the electrical connection between zinc particles. other conductive material with higher activity may also have this same anticorrosion mechanism.

However, practical applications face challenges due to difficulties in uniform dispersion and stability attributed from the structural characteristics and size effects of these nanomaterials (Chen et al., 2022; Green and Hersam, 2010; Zhao et al., 2022). At the same time, the high price of nanomaterials increases the overall cost of coatings, making them less viable for industrial production. Previous studies have shown that the use of functional materials (such as sulfonation) can solve the dispersion problem of graphene, but it can seriously reduce it conductivity, thereby diminishing the protective effectiveness of zinc rich coatings (Chen et al., 2022; Han et al., 2024; Li et al., 2021; Tian et al., 2021). Therefore, it is of great significance to identify better-dispersed and more economical conductive materials to serve as alternatives to zinc powder.

Loading graphene onto an appropriate carrier material is a feasible strategy to enhance its dispersion in coatings. Mica is a natural mineral comprised of various components, typically appearing as aggregated flakes or scales with a high aspect ratio and excellent toughness. Its layered structure remains intact during processing, offering significant durability. Additionally, mica exhibits outstanding chemical inertness, contributing to enhanced corrosion resistance in coatings against neutral salt spray, acids, and alkalis. These characteristics make mica an ideal carrier for conductive carbon-based materials such as graphene. Utilizing mica as a carrier enables graphene loading onto mica interfaces and promotes its uniform dispersion within the coating matrix (Dai et al., 2006). This approach is expected to enhance graphene’s distribution in coatings and optimize its exceptional conductivity, potentially improving the performance of graphene in zinc-enriched primers.

In addition to carbonous materials, conductive metals may also serve as viable substitutes for zinc. Among these, nickel powder stands out as a typical metal-based conductive filler, known for its fine particle size and excellent conductivity. Its unique three-dimensional chain structure facilitates the formation of an effective conductive network, and its good chemical stability, strong corrosion resistance, and even can be applied to a highly corrosive and elevated temperature environment. Nickel is commonly used as a buffer layer for surface coatings or as an alloy additive to enhance its corrosion resistance and conductivity (Bai et al., 2011). For example, Chao et al. (2023) developed a conductive coating on the surface of a magnesium alloy through micro-arc oxidation, incorporating nickel powder into an epoxy resin matrix. The results demonstrated that the coating containing nickel powder exhibited the lowest resistivity, with a value of 0.02 Ω·cm (Chao et al., 2023). When the nickel powder content reached 50 wt.%, the coating layer showcased excellent wear and corrosion resistance. These findings suggest that nickel holds potential as a partial substitute for zinc powder to achieve high corrosion resistance.

In this study, conductive graphene-mica powder and the conductive nickel were used to improve the corrosion performance of epoxy zinc-rich coatings. The conductive mica was prepared, and the effects of adding conductive nickel and graphene-doped mica on the properties of epoxy zinc-rich coatings were investigated. The morphologies and structures of the conductive additives and coating films were characterized, while the basic properties and protective performance of the coatings were comprehensively evaluated through electrochemical and salt spray tests.

## 2 Experimental

### 2.1 Materials

E51 epoxy resin (≥98%) was purchased from South Asia Epoxy Resin (Kunshan) Co., Ltd., Kunshan, China. The E44 epoxy resin (≥98%), mixed solvent and B18 active monomer were from Jiangsu Sanmu Chemical Co., Ltd., Jiangs, China. BYK163 dispersion, adhesion promoter 1,051, diego 680 antifoam, BYK410 polyamide wax and gas phase silica were purchased from Shanghai Haiyi Science and Trade Co., Ltd., Shanghai, China. Environmental zinc phosphate MT601 and conductive nickel BC-C were purchased from Chongqing Maitu Technology Co., Ltd. (Chongqing, China) and Shanghai Junjiang New Materials Sales Co., Ltd. (Shanghai, China), respectively. Zinc powder (industrial-grade) and graphene were from Sichuan Xinweiling Metal New Materials Co., Ltd. (Sichuan, China) and Suzhou Qualcomm New Material Co., Ltd. (Jiangsu, China), respectively. Leveling and amine modified curing agent 5,625 were purchased from Jiangsu Shisong New Materials Technology Co., Ltd., Jiangsu, China. Conductive Mica Powder was purchased from Shanghai Junjiang New Material, Shanghai, China.

The metallic substrates were Q235B steel panels, and the surface of steel bead blasting treatment, anchorage of 30 microns.

### 2.2 Preparation of the coating

#### 2.2.1 Preparation of conductive graphene-mica powder

14.8 g of deionized water, 5 g sodium dodecyl benzene sulfonate and 0.2 g graphene were emulsified for 40 min and continuously dispersed in the beaker by ultrasonic breaker (JP060S, Shenzhen Jieng Cleaning Equipment Co., LTD, China) for 60 min. 100 g mica was activated by soaking in 100 g 10% hydrochloric acid solution, after that was suspended in 8 g PVP and 15 g PVA solution. 20 g graphene emulsion and 123 g mica suspension were then mixed for 20 min at 60 r/min in a three-mouth flask regulating pH to 2.5 by 10% HCl. Under constant stirring, a certain concentration of the mixture of 4 g stannic chloride and 0.5 g antimonous chloride was added, maintaining pH at 2.2 and the temperature between 70°C–80°C. After 30 min, the obtained solid was washed with deionized water. The filter cake was dried at 120°C for 4 h and calcined at 600°C–700°C for 30 min in the oxygen atmosphere. Finally, the conductive graphene-mica powder was prepared.

#### 2.2.2 Preparation of epoxy zinc-rich coating containing conductive particles

The coating is a two-component epoxy coating composed of component A and component B. The preparation process of components A was as follows: E51, E44, dispersion agent and defoamer were added subsequently, followed by stirring at 400 rpm for 10 min, Next, polyamide wax, fumed silica and eco-friendly zinc phosphate were added in order, and the mixture was stirred at 900 rpm for 20 min, and subsequently ground to 50 microns using the grinder (LSM-2.2D, Shanghai Tianchen Co., Ltd. Shanghai, China). Conductive nickel or conductive graphene-mica was added under stirring at 800 rpm. After 20 min, the zinc powder, adhesion promoter and leveling agent were added in turn while stirring, and component A of paint was successfully prepared. The percentage of raw materials in components A of epoxy zinc-rich coatings with Ni and conductive graphene-mica are listed in Tables 1, 2, respectively. Component B of paint was prepared by evenly mixing the modified amine curing agent, amine promoter with solvent at 800 rpm for 10 min. The percentage of raw materials in components B of epoxy zinc-rich coatings was shown in Table 3.

**TABLE 1:** Percentage of components A of epoxy zinc-rich coatings added Ni.

Raw material	Ni5	Ni10	Ni15
E44 epoxy resin, %	11	11	11
E51 epoxy resin, %	1	1	1
Dispersant, %	1	1	1
Dimethylbenzene, %	6	6	6
n-butyl alcohol, %	2	2	2
Polyamide wax, %	0.3	0.3	0.3
Gas phase silica, %	0.2	0.2	0.2
Eco-friendly zinc phosphate, %	8	8	8
Conductive nickel, %	5	10	15
Zinc powder, %	65	60	55
Epoxy facilitator, %	0.5	0.5	0.5
Total, %	100	100	100

**TABLE 2:** Percentage of components A of epoxy zinc-rich coatings added conductive graphene-mica.

Raw material	GM15	GM20	GM25
E44 epoxy resin, %	12	12	12
E51 epoxy resin, %	1	1	1
Dispersant, %	2	2	3
Dimethylbenzene, %	7	10	10
n-butyl alcohol, %	2	2	3
Polyamide wax, %	0.3	0.3	0.3
gas phase silica, %	0.2	0.2	0.2
Tribasic zinc phosphate, %	5	2	0
Conductive graphene-mica, %	15	20	25
Zinc powder, %	55	50	45
Epoxy facilitator, %	0.5	0.5	0.5
Total, %	100	100	100

**TABLE 3:** Percentage of components B of epoxy zinc-rich coatings.

Raw material	Percentage
Modified amine curing agent, %	50
Amide promoters, %	2
Solvent, %	48
Total, %	100

#### 2.2.3 Preparation of coating

Component A and Component B were mixed at the weight ratio of 10:1, and spraying viscosity was adjusted to 30-40S using solvent. The mixture was then sprayed on the surface of horse mouth iron and carbon steel, respectively. The coating sample with the thickness of 20–30 µm was applied to test the conventional performance after placed in a 25°C incubator for 48 h. The carbon steel with the coating thickness of 90–100 µm was used for salt spray resistance and chemical resistance tests.

### 2.3 Testing and characterization

#### 2.3.1 Morphological characterization

The structure of materials was tested by FTIR (DX-2700X, American Platinum Elmer, Inc., United States) and XRD (DX-2700X). The microtopography of the filler and the coating surface was observed using a scanning electron microscope (SEM, TESCAN VEGA3SBU). The conductive nickel and graphene distribution of conductive graphene-mica in coating was analyzed by Energy Dispersive Spectrometer (XPS, Axis Utltra DD, Kratos, Britain).

#### 2.3.2 Coating testing

The impedance test of the coating was performed with the electrochemical workstation CHI660e (Shanghai Chenhua). And the impedance test was performed for up to 1,200 h. The electrochemical impedance test is a three-electrode system, the saturated calomel electrode is the reference electrode, the platinum electrode is the opposite electrode, the working electrode is a coated metal sample, and the test solution is 3.5 wt.% sodium chloride solution. The measured electrochemical data were analyzed by fitting using ZSimpWin software.

The neutral salt spray resistance test was conducted using the salt spray machine of Sichuan Chuangbei Technology Co., Ltd. for 2,000 h. The corrosion progression was acquired by measuring the average width of unilateral corrosion expansion normal to the scribe direction, which was calculated by subtracting the observed corrosion width of eight rusted areas by the scribe width and dividing by two. The test standard for bending performance was ISO 1519 using cylinder bending tester from the Precision Instruments (Guangzhou) Co., Ltd. The test standard for impact resistance was ISO 6272 using the impact instrument from the Precision Instruments (Guangzhou) Co., Ltd. The test standard for scratch test was ISO 4624. According to the test standard, a grid pattern of scratches was created on the surface using a knife. A piece of adhesive tape was then applied to the center of the grids and smoothed down firmly. The tape was pulled away smoothly, and the extent of film detachment within the grids was observed and evaluated to determine the adhesion quality of the film.

## 3 Result and discussion

### 3.1 Morphology analysis of conductive filler and coatings

**FIGURE 1:**
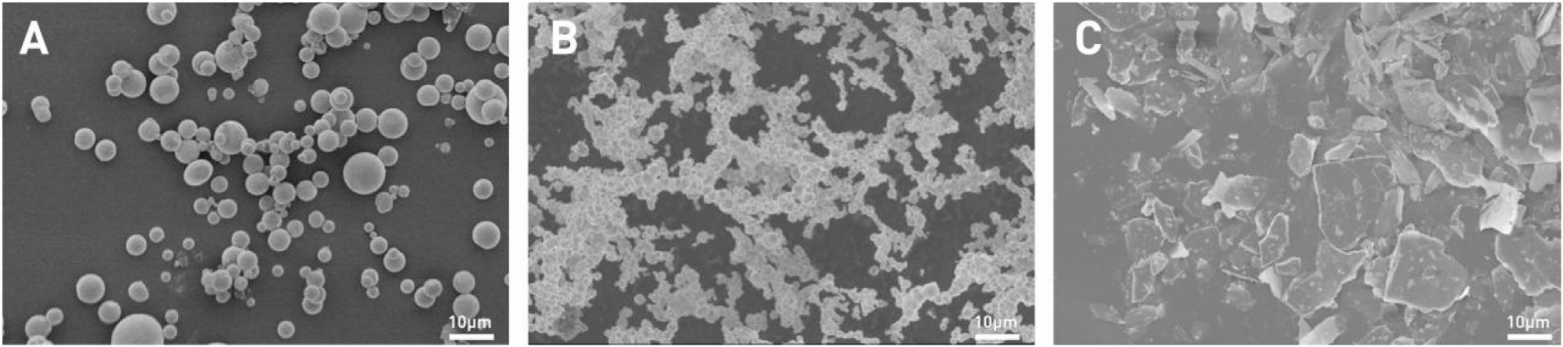
SEM of conductive fillers (**(A)**-zinc powder; **(B)**-conductive nickel; **(C)**-doped graphene conductive mica).

Figure 1 shows the SEM photos of zinc powder, conductive nickel and conductive graphene-mica. From the SEM images, it can be observed that the particle size of zinc powder ranges from approximately 2 to 10 μm. Conductive nickel appears as microspheres with uniform size of approximately 2–3 μm. These microspheres are interconnected, forming a bead-like chain structure, which facilitates the creation of a conductive network. Conductive mica, on the other hand, exhibits a two-dimensional sheet-like morphology with planar dimensions ranging from 5 to 20 μm. The larger surface of the mica is advantageous for enhancing the connectivity of zinc powder particles.

**FIGURE 2:**
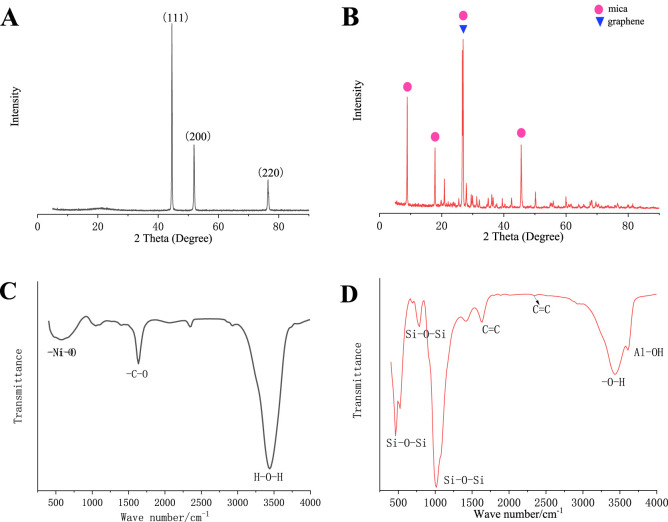
**(A)** XRD of conductive nickel, **(B)** XRD of conductive graphene-mica, **(C)** FTIR of conductive nickel and **(D)** FTIR of conductive graphene-mica.

As shown in Figure 2A, the crystal configuration of the nickel sample is the surface center cubic structure, with the most intense peaks (111), (200), (220), consistent with Ni (PDF 04-0850). And there are no impurity peaks in the spectra, indicating that the nano nickel particles have high purity, single phase composition, and are not significantly oxidized. Figure 2B shows the XRD patterns of doped graphene conductive mica. It can be seen that doped graphene conductive mica displays a strong peak around 26°, which may belong to the (002) of graphite. Figure 2C shows the FTIR patterns of conductive nickel. Nickel metal has low infrared activity and no obvious infrared absorption peak. The weak absorption peek at low wave number of around 500 cm^−1^ is assigned to nickel oxide, indicating a slight oxidation on the nickel surface. The observed absorption bands 3,400 cm^−1^ and 1,700–1,600 cm^−1^ are attributed to the adsorbed water and other oxides species. Figure 2D shows the FTIR patterns of doped graphene conductive mica. The peak at 1,060–1,050 cm^−1^ is caused by the vibration of the C-C single bonds between the carbon atoms in graphene. Graphene-specific absorption peaks (1,620–1,600 cm^−1^, 2,200–2,100 cm^−1^) versus the C=C double bond vibration between carbon atoms in graphene. For mica, it has a moderate intensity absorption peak near 3,620 cm^−1^ in the high frequency region, belonging to the vibration of Al-O-H. Absorption peaks at 1,020 cm^−1^, 770 cm^−1^ and 520 cm^−1^ are attributed to Si-O-Si vibration.

**FIGURE 3:**
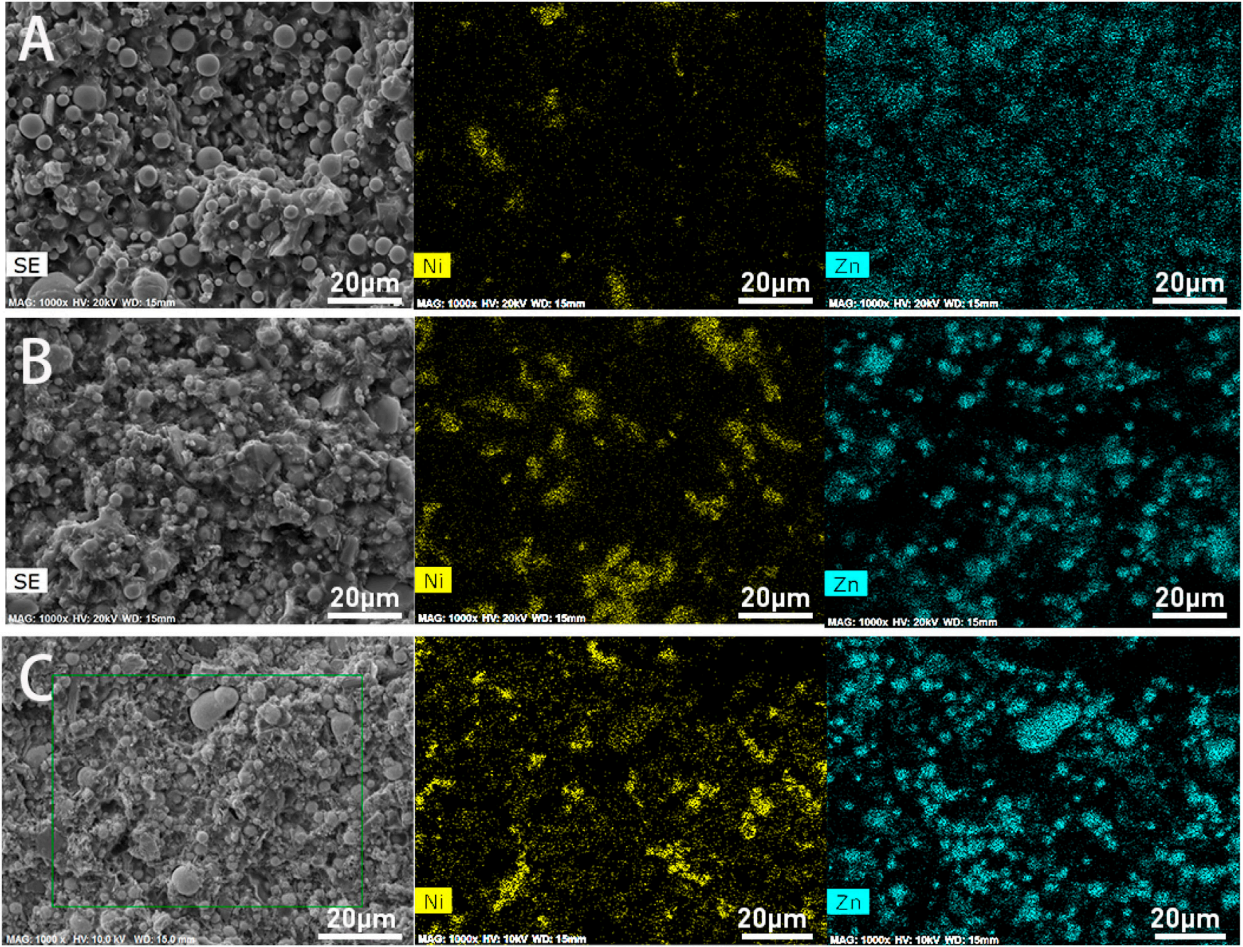
Distribution of Ni and Zn elements in the coating with conductive nickel powder (**(A)**-Ni5; **(B)**-Ni10; **(C)**-Ni15).

Figure 3 is the morphology and element distribution of the three kinds of epoxy zinc-rich coatings with different conductive nickel content. As shown in Figure 3A, distinct zinc powder particles can be observed, with zinc elements evenly dispersed throughout the coating. However, due to the low nickel content, a significant number of zinc particles remain isolated, and the connection between zinc and nickel is incomplete. In Figure 3B, as the nickel content increases, zinc and nickel powders become more intermingled, leaving almost no isolated zinc particles. The nickel powder effectively serves as a bridging agent, connecting the zinc particles. Although some aggregation of nickel powder occurs, the overall distribution of both nickel and zinc remains relatively uniform. With a further increase in nickel content and a corresponding decrease in zinc content, Figure 3C shows that nickel powder aggregation becomes more pronounced. A large portion of the zinc powder is embedded within the nickel, making zinc particles almost undetectable except for a few larger ones. This severe aggregation of nickel powder and the encapsulation of zinc particles hinder the connectivity of zinc and compromise its sacrificial anode function.

**FIGURE 4:**
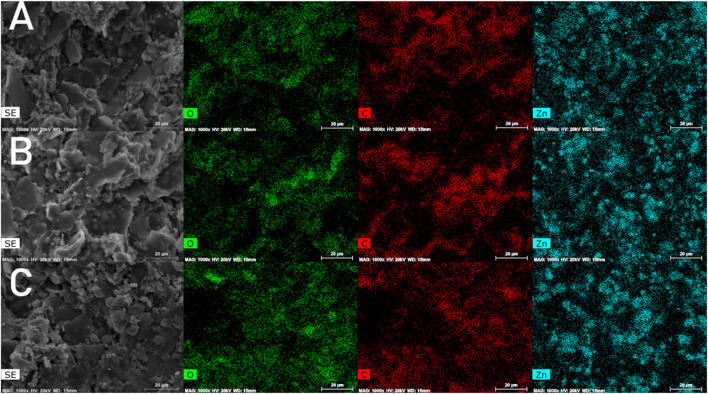
Distribution of C element, O element and Zn element in doped graphene conductive mica coating (**(A)**-GM15; **(B)**-GM20; **(C)**-GM25).

The morphology and elemental analysis of the epoxy zinc-rich coating containing conductive graphene-mica are presented in Figure 4. The images reveal that the conductive graphene-mica, with its lamellar structure, is uniformly dispersed throughout the coating and is neatly aligned. The elemental distribution analysis shows that zinc is uniformly dispersed within the coating, maintaining an orderly arrangement. This indicates that the incorporation of conductive graphene-mica does not cause zinc powder aggregation in the epoxy coating. As the amount of conductive graphene-mica increases, its sheet-like structure becomes more prominent. Better connection of zinc is expected at higher content of conductive mica.

### 3.2 Corrosion resistance of coating

#### 3.2.1 Salt spray resistance of the coating

**FIGURE 5:**
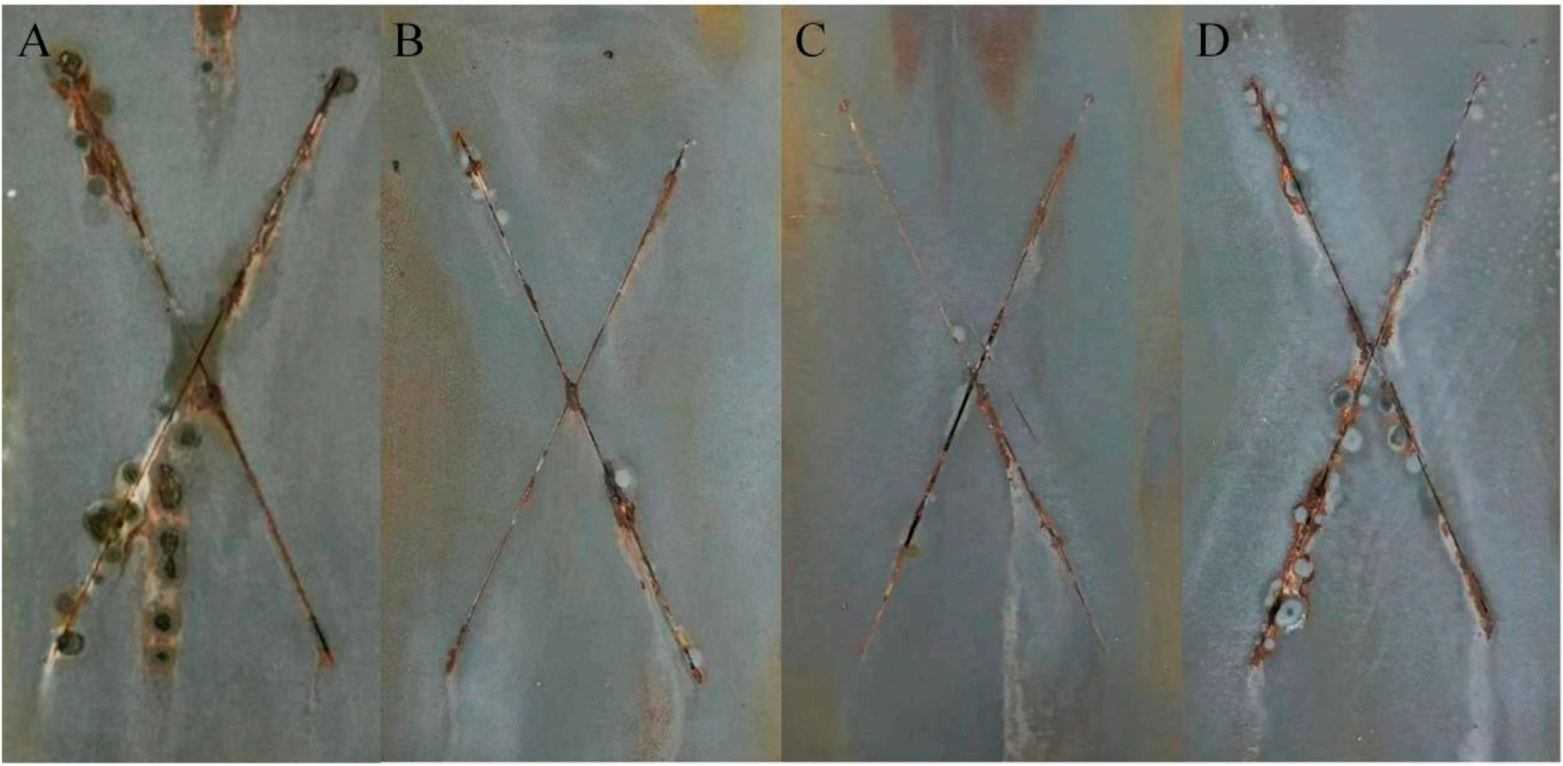
2,000 h salt fog resistance photos of conductive nickel-containing epoxy zinc-rich coating **(A)** Zn70 **(B)** Ni5 **(C)** Ni10 **(D)** Ni15.

**FIGURE 6:**
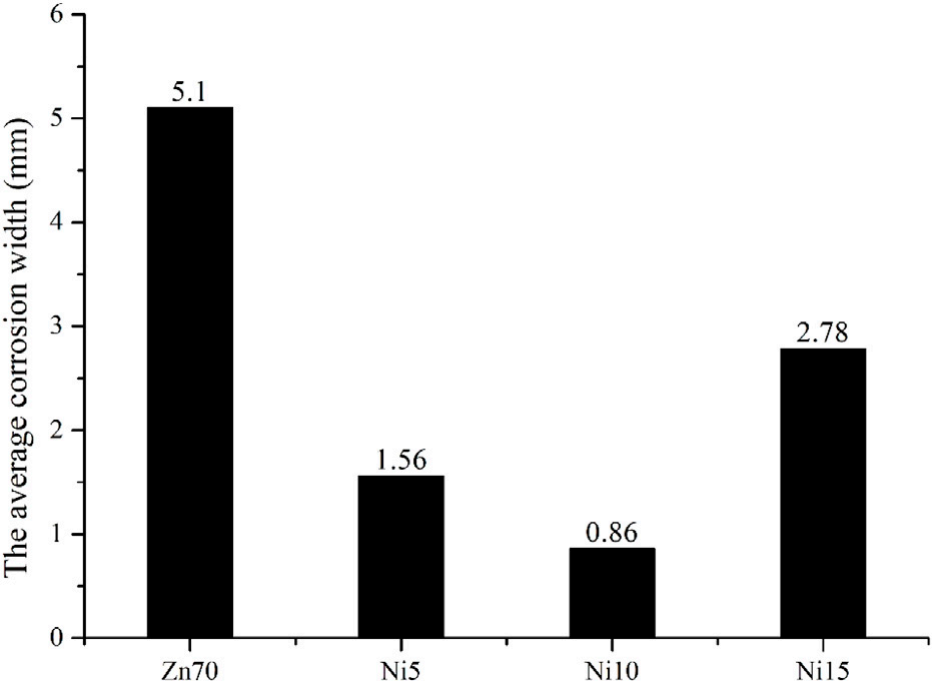
The average unilateral corrosion width of conductive nickel-containing epoxy zinc-rich coating.

Figure 5 is a macro photo of 2,000 h salt spray of epoxy zinc-rich coatings with different conductive nickel contents and Figure 6 lists the unilateral corrosion width results. It can be seen that the epoxy zinc-rich coating with 70 wt.% Zn has substantial rust spots after 2,000 h of salt spray testing. The average unilateral corrosion expansion width at the scratch area is as high as 5.10 mm. When 10 wt.% conductive nickel is added, coating has fewer rust spots and the lowest corrosion with of 0.86 mm. With 5 wt.% conductive nickel coating has some rust and blisters, while the epoxy zinc-rich coating with 15% conductive experiences severe rusting and blistering. Therefore, the optimal salt spray resistance is achieved with epoxy zinc-rich coating with 10% conductive nickel. The reason may be that the nickel content affects its dispersion in the coating, which affects the corrosion resistance of the coating. When the nickel content is relatively low, it is insufficient to compensate for the reduction in zinc powder, failing to form an effective conductive pathway, which results in suboptimal corrosion resistance. Conversely, when the nickel content is too high, it disrupts the dispersion of zinc powder within the coating, leading to agglomeration that negatively impacts the coating’s corrosion resistance. Therefore, when the nickel content is set to 10%, the amount is more balanced, resulting in the optimal corrosion resistance.

**FIGURE 7:**
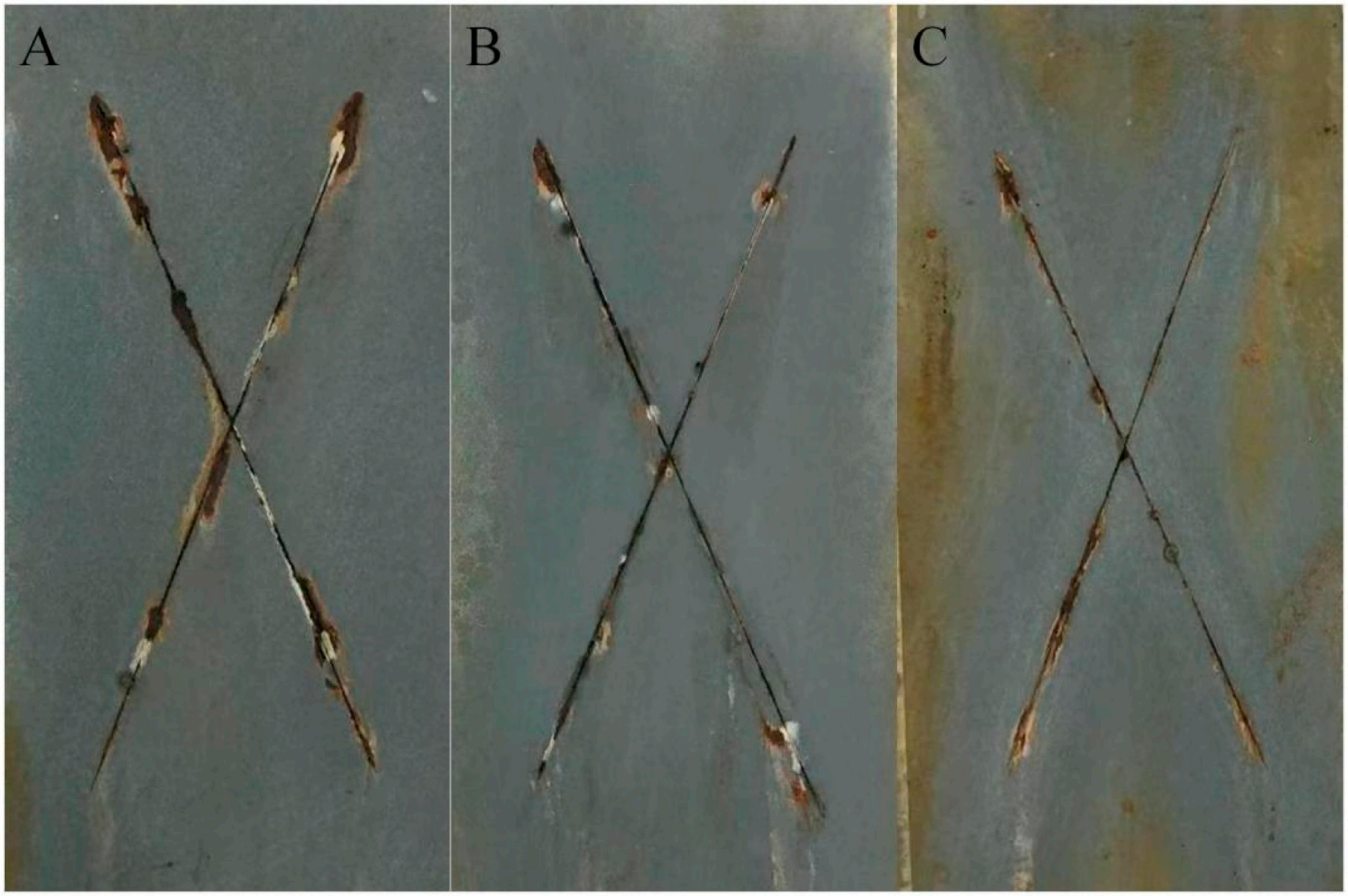
Photos of graphene-modified conductive mica epoxy zinc-rich coating resistant to salt spray for 2,000 h **(A)** GM15 **(B)** GM20 **(C)** GM25.

**FIGURE 8:**
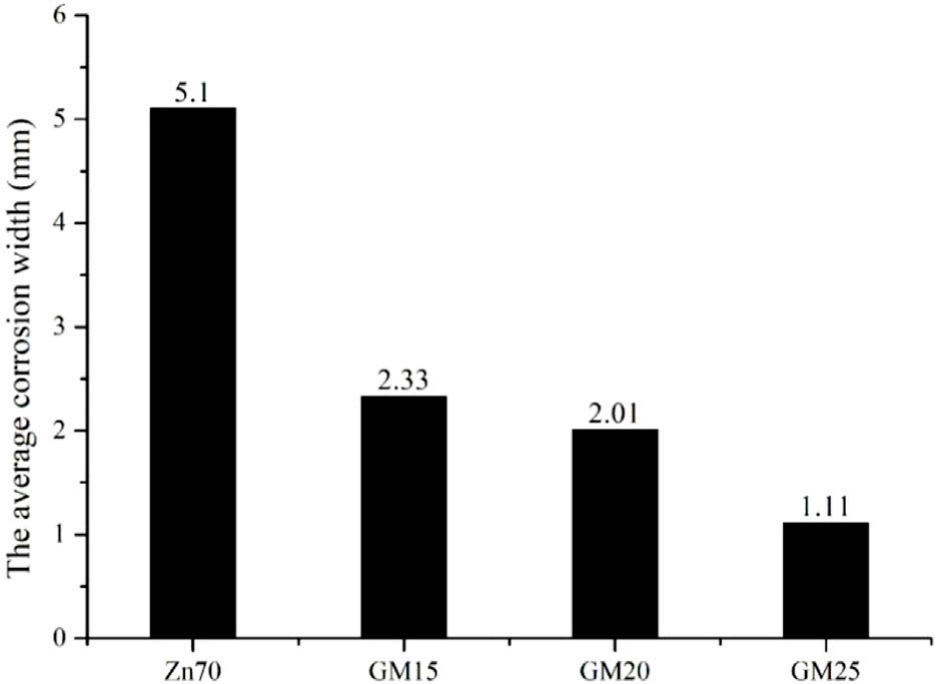
The average corrosion width of conductive graphene-mica epoxy zinc-rich coating.

Figure 7 shows the macroscopic photos of different epoxy zinc-rich coatings incorporating different levels of conductive graphene-mica at 2,000 h of salt spray exposure, while Figure 8 provides the quantitative corrosion results. It is evident that coatings with 15 wt.% and 20 wt.% conductive graphene-mica show signs of corrosion with the unilateral corrosion widths are 2.33 and 2.01 mm, respectively, while the coating with 25 wt.% graphene-modified conductive mica remains almost corrosion-free with the lowest corrosion width of the three coating samples. According to the analysis, when the content of conductive graphene-mica is small, it cannot be fully distributed in the epoxy resin, so that the corrosion prevention ability of conductive graphene-mica cannot be fully reflected. As contrast, when the amount of conductive graphene-mica added increases, the bridging effect is enhanced ensuring a better zinc conductive network and improved sacrificial anode behavior, and barrier effect also increase, making it more difficult for the corrosion medium to reach the metal surface, thus slowing down the corrosion rate of the metal. Therefore, increasing the amount of conductive graphene-mica enhances the corrosion resistance of the zinc-rich coating.

Both conductive nickel and graphene-modified mica demonstrate the ability to reduce zinc content, while maintaining superior anticorrosion ability, as shown in the corrosion morphologies of different coatings (Figures 5, 7). The white part is mainly ZnO or Zn_5_(CO_3_)_2_(OH)_6_ from the oxidation of zinc. The mechanism by which conductive fillers reduce the zinc powder content in zinc-rich coatings lies in their ability to maintain or enhance the electrical conductivity of the coating matrix while decreasing the reliance on zinc particles for percolation pathways. Zinc-rich coatings primarily protect substrates through sacrificial anode behavior, where zinc particles corrode preferentially to the steel substrate, forming a conductive network that facilitates electron transfer. Conductive nickel and graphene-modified mica supplement this network by bridging zinc particles. This reduces the critical zinc concentration required to achieve electrical percolation, thereby lowering the overall zinc content without compromising the coating’s cathodic protection capability. Additionally, conductive fillers, especially graphene-modified mica can also improve barrier properties prolonging the coating’s protective lifespan (Wang et al., 2024; Al-Nafai et al., 2025).

Recent literatures further corroborate our findings. For instance, a 2024 study from Zhonghua Chen et al. reported that integrating nickel with reduced graphene oxide and nitrogen-doped porous carbon in zinc-based coatings enabled a significant reduction in zinc content while simultaneously enhancing corrosion resistance (Chen et al., 2019). Likewise, graphene-based conductive fillers have been shown to lower the zinc content to below 50 wt% while maintaining or even surpassing the anticorrosion performance of traditional 70 wt% Zn coatings. Our previous study also showed that polypyrrole modified conductive mica could decrease the zinc content by 14% while maintaining a promising anticorrosion performance (Yuxing et al., 2022). The current results are in excellent agreement with these findings, as the incorporation of nickel reduces the zinc content to 60 wt% and the addition of graphene-modified conductive mica decreases it to as low as 45 wt%, all while delivering superior corrosion protection.

#### 3.2.2 Electrochemical impedance analysis

**FIGURE 9:**
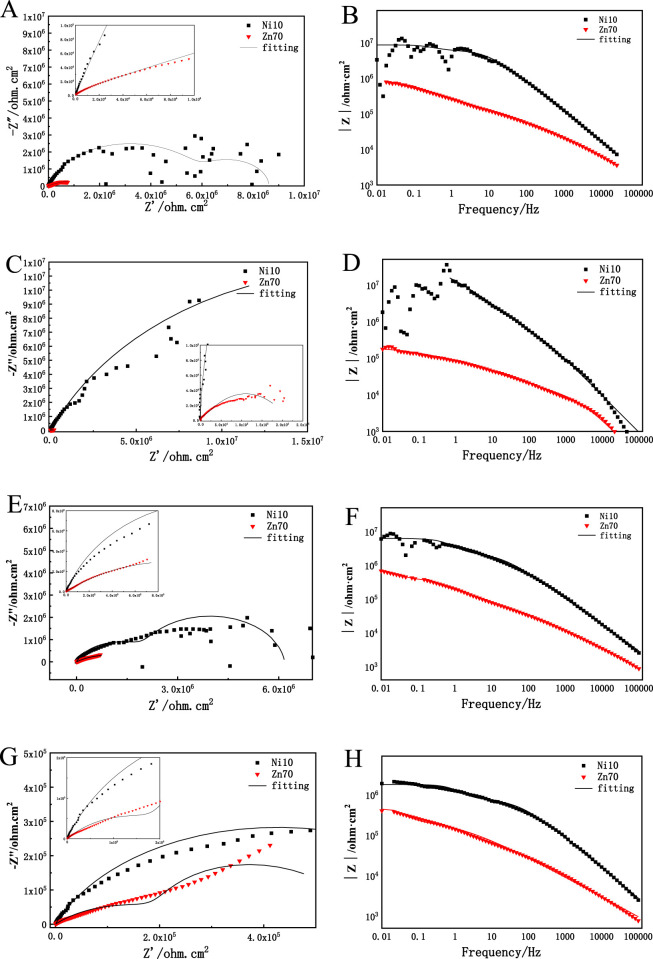
Impedance spectra of three kinds of epoxy zinc-rich coatings in 3.5% NaCl solution at different times **(A, B)** 15 h; **(C, D)** 100 h; **(E, F)** 600 h; **(G, H)** 1,200 h.

Currently, the electrochemical mechanism of nickel-assisted zinc-rich coatings has not been investigated, thus, this study also investigates the electrochemical performance of the coating. The salt spray test results indicates that Ni10 coatings have excellent salt spray resistance. In order to further analyze the performance of the coating, the pure epoxy zinc-rich coating and conductive nickel epoxy zinc-rich coating were selected for EIS. The test solution was 3.5 wt.% NaCl solution, as shown in Figure 9. The high impedance value of Ni10 indicates its superior barrier properties, effectively suppressing bulk electrolyte penetration. However, this characteristic notably amplifies the sensitivity of low-frequency impedance signals to localized micro-defects. At elevated coating impedance levels, even partially unmitigated micro-corrosion regions can induce significant fluctuations in the low-frequency region due to localized electrochemical activity, which becomes disproportionately detectable in high-impedance systems, especially at the early stage of the immersion. For better understanding the mechanism, the impedance value of Bode diagram at low frequency (0.01 Hz) can be used as a reference for evaluating the protective performance of coatings (Liu et al., 2025; Ke et al., 2023). Table 4 show quantitative data for impedance values of epoxy zinc-rich coatings at 0.01 Hz. After 1,200 h immersion, conductive nickel epoxy zinc-rich coating has a maximum impedance value of 1.88 × 10^6^ Ω·cm^2^ at the frequency of 0.01 Hz (Table 4). It is about five times of that of the original Zn 70 coatings, which indicates that the conductive nickel epoxy zinc-rich coating has better protective performance. In the Nyquist diagram, a large capacitance arc radius corresponds to excellent corrosion resistance. When the corrosive medium does not reach the coating/ substrate interface, a single capacitance arc appears on the Nyquist diagram. If the corrosive medium reaches the substrate, there are two capacitive arcs (Conradi et al., 2014; Brug et al., 1984; Mondal et al., 2016; Wu et al., 2019). It can be seen from the Nyquist diagram of Figure 9 that the conductive nickel epoxy zinc-rich coating has the largest impedance arc and the best protective performance during the whole immersion process.

**TABLE 4:** Quantitative data for impedance values of epoxy zinc-rich coatings at 0.01 Hz.

Immersion time	|Z|/ohm·cm^2^(Zn70)	|Z|/ohm·cm^2^(Ni10)
15 h	9.02E+05	8.62E+06
600 h	6.89E+05	5.93E+06
1,200 h	3.99E+05	1.88E+06

**FIGURE 10:**
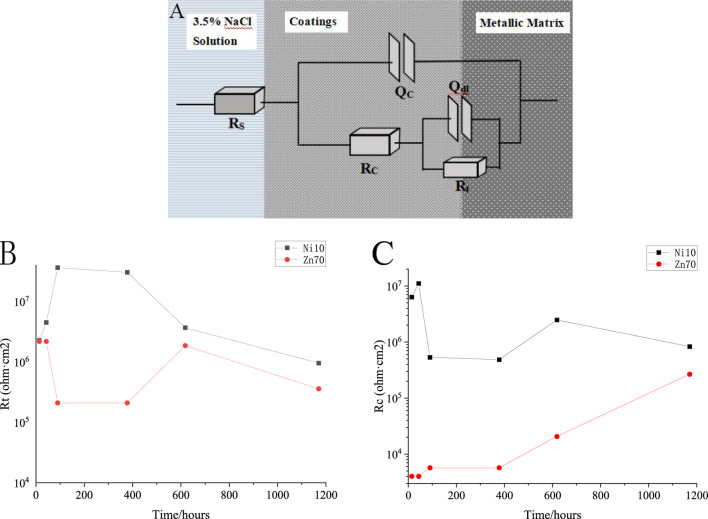
**(A)** Equivalent circuit diagram; **(B)** Curve of *R*_*t*_ changing with soaking time; **(C)** Curve of *R*_*c*_ changing with soaking time.

The corrosion process and properties of the coating were further analyzed by fitting the EIS results with Zsimpwin software. The circuit corrosion model at each stage in this work is shown in Figure 10A. Among them, *R*_*s*_ represents solution resistance, *Q*_*c*_ and *R*_*c*_ represent coating capacitance and coating resistance respectively, *Q*_*dl*_ and *R*_*t*_ represent electric double layer capacitance under coating and reaction resistance of metal under coating respectively. Curve of Rt and Rc changing with soaking time is shown in Figures 10B,C, respectively. In the EIS test, the higher the resistance (*R*_*c*_) of the coating, the better the shielding performance of the coating. The higher the *R*_*t*_ value, the more difficult the corrosion reaction of the metal substrate under the coating. It is found that the conductive nickel epoxy zinc-rich coating has good coating resistance and shielding performance during the 1,200-h immersion, which provides good protection for the metal. In the early stage of corrosion protection, the corrosive medium gradually penetrates into the matrix, resulting in a decrease in *R*_*c*_ value. However, the shielding performance of the coating was subsequently improved, which may be due to the fact that the entire zinc powder material in parallel and in series forms a huge anode network, so that all zinc powders achieve network interconnection. When the corrosive medium invades the coating, the weakest link of the coating cannot be found, which greatly enhances the shielding effectiveness of the coating. After partial oxidation of zinc particles, stable conductive additives can maintain electron transfer pathways between metallic phases through physical contact with unoxidized zinc particles, thereby ensuring the continuity of the conductive network to maintain the protection effect.

### 3.3 Comprehensive performance of coating

Table 5 is the basic performance test results of epoxy zinc-rich coating, conductive nickel epoxy zinc-rich coating and conductive graphene-mica coating. It was found that the addition of conductive nickel and conductive graphene-mica powder in the epoxy zinc-rich coating will not affect the basic properties of the coating, which provides data support for the industrialization of the coating.

**TABLE 5:** Basic performance test results of coating.

Tests	70% zinc-rich coating Zn70	Conductive nickel zinc-rich coating			conductive graphene-mica zinc-rich coating		
		Ni5	Ni10	Ni15	GM15	GM20	GM25
Bending performance(mm)	1	1	1	1	1	1	1
Impact resistance(cm)	50	50	50	50	50	50	50
Scratch test (grade)	1	1	1	1	1	1	1

## 4 Conclusion

In this paper, conductive graphene-mica was prepared, and the conductive graphene-mica and nickel were incorporated to enhance the corrosion protection of epoxy zinc-rich coatings. The addition of conductive nickel or mica creates an interpenetrating network within the coating, significantly improving its corrosion resistance. The optimal epoxy zinc-rich coatings with nickel powder or conductive graphene-mica show less corrosion marks after 2,000 h of salt spray at reduced zinc content of 60 wt% or 45 wt%, respectively, compared to conventional coatings with 70% pure zinc powder (Zn70), demonstrating outstanding salt spray resistance and superior anti-corrosion performance. Electrochemical impedance spectroscopy (EIS) revealed that the |Z|0.01 Hz value of the nickel epoxy zinc-rich coatings is almost one order of magnitude higher than that of Zn70, verifying a robust barrier effect. Additionally, the mechanical strength of the coatings with conductive additives was comparable to that of coatings with pure zinc, maintaining excellent overall performance. This paper develops a conductive mica material and explores the metal conductive candidate, providing a scalable strategy to efficiently reduce zinc content in epoxy zinc-rich coating. Future research will focus on designing hierarchical conductive composites and optimizing carrier materials to further minimize zinc dependency while maximizing corrosion protection efficiency, and optimizing material formulations through strategic substitution of high zinc content with minimal conductive additives, thereby achieving enhanced cost-effectiveness for industrial-scale applications.

## Data Availability

The original contributions presented in the study are included in the article/supplementary material, further inquiries can be directed to the corresponding authors.

## References

[B1] Al-Nafai I. Rzeszutek K. Lyon S. Jones C. Beaumont D. (2025). How aluminium additions improve the performance of zinc‐rich organic coatings. Mater. Corros. 76, 53–70. 10.1002/maco.202414529

[B2] Bai C.-Y. Lee J. L. Wen T. M. Hou K. H. Wu M. S. Ger M. D. (2011). The characteristics of chromized 1020 steel with electrical discharge machining and Ni electroplating pretreatments. Appl. Surf. Sci. 257 (8), 3529–3537. 10.1016/j.apsusc.2010.11.070

[B3] Brug G. J. van den Eeden A. L. G. Sluyters-Rehbach M. Sluyters J. (1984). The analysis of electrode impedances complicated by the presence of a constant phase element. J. Electroanal. Chem. 176, 275–295. 10.1016/0368-1874(84)83477-2

[B4] Chao Z. Chao W. Bo J. Renguo S. (2023). Preparation and research of AZ31 magnesium alloy micro-arc oxidation/epoxy rensin-nickel powder conductive coating. Mater. Prot. 56 (8), 64–69.

[B5] Chen Z. Cai Y. Lu Y. Cao Q. Lv P. Zhang Y. (2022). Preparation and performance study of carboxy-functionalized graphene oxide composite polyaniline modified water-based epoxy zinc-rich coatings. Coatings 12 (6), 824. 10.3390/coatings12060824

[B6] Chen Z. Qing L. Chang H. (2019). The effect of the oxidation degree and content of graphene on the anticorrosion performance of waterborne epoxy zinc‐rich coatings. Paint Coatings Industry Chin. 49 (06). 10.12020/j.issn.0253-4312.2019.6.35

[B7] Conradi M. Kocijan A. Kek-Merl D. Zorko M. Verpoest I. (2014). Mechanical and anticorrosion properties of nanosilica-filled epoxy-resin composite coatings. Appl. Surf. Sci. 292, 432–437. 10.1016/j.apsusc.2013.11.155

[B8] Dai H. Li H. Wang F. (2006). Electroless Ni–P coating preparation of conductive mica powder by a modified activation process. Appl. Surf. Sci. 253 (5), 2474–2480. 10.1016/j.apsusc.2006.05.010

[B9] Fang X. Yuan Y. Wang Q. Ji C. Wu Y. Liu H. (2024). Effect of zinc powder reduced graphene oxide on the corrosion resistance of waterborne inorganic zinc-rich coatings. Coatings 14 (10), 1321. 10.3390/coatings14101321

[B10] Gergely A. Pászti Z. Mihály J. Drotár E. Török T. (2014). Galvanic function of zinc-rich coatings facilitated by percolating structure of the carbon nanotubes. Part II: protection properties and mechanism of the hybrid coatings. Prog. Org. Coatings 77 (2), 412–424. 10.1016/j.porgcoat.2013.11.004

[B11] Green A. A. Hersam M. C. (2010). Emerging methods for producing monodisperse graphene dispersions. J. Phys. Chem. Lett. 1 (2), 544–549. 10.1021/jz900235f 20657758 PMC2908411

[B12] Han X. Kong J. Zhang H. Zhao Y. Zheng Y. Wei C. (2024). Triglycerides mediate the influence of body mass index on non-alcoholic fatty liver disease in a non-obese Chinese population with normal low-density lipoprotein cholesterol levels. Prog. Org. Coatings 17, 191–200. 10.1159/000536447 PMC10987190 38266508

[B13] Hayatdavoudi H. Rahsepar M. (2017). A mechanistic study of the enhanced cathodic protection performance of graphene-reinforced zinc rich nanocomposite coating for corrosion protection of carbon steel substrate. J. Alloys Compd. 727, 1148–1156. 10.1016/j.jallcom.2017.08.250

[B14] He D. Wang L. Yang Z. Sun W. Feng Y. Xu K. (2024). A New understanding of graphene influencing the protective performance of zinc-rich coatings. Industrial Eng. Chem. Res. 63 (17), 7661–7672. 10.1021/acs.iecr.4c00089

[B15] Ke C. Liu J. Liu Y. Li Z. Du T. Wu Y. (2023). Photothermal MOF-Based multifunctional coating with passive and active protection synergy. ACS Appl. Eng. Mater. 1 (3), 1058–1068. 10.1021/acsaenm.3c00027

[B16] Kratochvílová I. Ashcheulov P. karohlíd J. Koda R. Steinbrück M. (2019). Zr alloy protection against high-temperature oxidation: coating by a double-layered structure with active and passive functional properties. Corros. Sci., 108270. 10.1016/j.corsci.2019.108270

[B17] Lan Y.-X. Cho Y. C. Liu W. R. Wong W. T. Sun C. F. Yeh J. M. (2023). Small-load rGO as partial replacement for the large amount of zinc dust in epoxy zinc-rich composites applied in heavy-duty anticorrosion coatings. Prog. Org. Coatings 175, 107332. 10.1016/j.porgcoat.2022.107332

[B18] Li H. Xue C. Gao L. Wang X. Wei H. Nan H. (2021). Labyrinthine structure anticorrosive water-based composite coatings. Prog. Org. Coatings 150, 105974. 10.1016/j.porgcoat.2020.105974

[B19] Liu J. X. Fang Y. Ou Y. Shi X. Zhang Y. Chen Q. (2025). Synergistic anti-corrosion and anti-wear of epoxy coating functionalized with inhibitor-loaded graphene oxide nanoribbons. J. Mater. Sci. Technol. 220, 140–149. 10.1016/j.jmst.2024.08.063

[B20] Mondal J. Marques A. Aarik L. Kozlova J. Simões A. Sammelselg V. (2016). Development of a thin ceramic-graphene nanolaminate coating for corrosion protection of stainless steel. J. Environ. Degrad. Mater. its Control 105 (Apr), 161–169. 10.1016/j.corsci.2016.01.013

[B21] Park S. M. Shon M. (2015). Effects of multi-walled carbon nano tubes on corrosion protection of zinc rich epoxy resin coating. J. Industrial Eng. Chem. 21, 1258–1264. 10.1016/j.jiec.2014.05.042

[B22] Ramezanzadeh B. Mohamadzadeh Moghadam M. Shohani N. Mahdavian M. (2017). Effects of highly crystalline and conductive polyaniline/graphene oxide composites on the corrosion protection performance of a zinc-rich epoxy coating. Chem. Eng. J. 320, 363–375. 10.1016/j.cej.2017.03.061

[B23] Son D. Yang C. Chen J. Qiao X. Zhang S. (2024). Enhanced protective performance of carbon nanotube-reinforced waterborne epoxy zinc-rich coatings for corrosion protection of steel structures. Coatings 14 (12), 1493. 10.3390/coatings14121493

[B24] Tian Y. Bi Z. Cui G. (2021). Study on the corrosion resistance of graphene oxide-based epoxy zinc-rich coatings. Polym. (Basel) 13 (10), 1657. 10.3390/polym13101657 PMC8160921 34069742

[B25] Wang X. Liang X. Wang B. Guo C. q. Zhang S. g. Yang K. (2024). Corrosion resistance of graphene/basalt flake modified waterborne epoxy zinc-rich primer. Pigment Resin Technol. 56 (6), 786–796. 10.1108/prt-08-2022-0101

[B26] Wang X. Lv J. Ding R. Gui T. j. Sun M. L. (2020). Application of EIS and transmission line model to study the effect of arrangement of graphene on electromagnetic shielding and cathodic protection performance of zinc-rich waterborne epoxy coatings. Int. J. Electrochem. Sci. 15 (5), 4089–4101. 10.20964/2020.05.60

[B27] Wu Y. Jingjing Z. Wenjie W. Bin L. Guang M. (2019). Investigating the anti-corrosion behaviors of the waterborne epoxy composite coatings with barrier and inhibition roles on mild steel. Prog. Org. Coatings. Inter. Rev. J. 133. 8–18. 10.1016/j.porgcoat.2019.04.028

[B28] Yuxing B. Jin X. Xie J. Lv X. Guo T. Zhang L. (2022). Fabrication of a conductive additive for the anticorrosion enhancement of zinc-rich epoxy coatings. Coatings 12 (10), 1406. 10.3390/coatings12101406

[B29] Zhang J. Zheng Y. (2023). Corrosion protection performance of graphene-modified zinc-rich epoxy coatings under high-temperature and high-concentration NaCl solution. J. Mater. Sci. 58 (30), 12202–12220. 10.1007/s10853-023-08621-1

[B30] Zhao X. Qi Y. Zhang Z. Li M. (2022). Effect of regularly arranged reduced graphene oxide on the anti-corrosion performance of waterborne silicate zinc-rich coatings. Corros. Eng. Sci. Technol. 58 (1), 61–72. 10.1080/1478422x.2022.2140248

[B31] Zhou S. Zhao L. Liang Z. Liu S. Li Y. Liu S. (2019). Indoleamine 2,3-dioxygenase 1 and programmed cell death-ligand 1 Co-expression predicts poor pathologic response and recurrence in esophageal squamous cell carcinoma after neoadjuvant chemoradiotherapy. Mater. Des. 11, 169. 10.3390/cancers11020169 PMC6406509 30717285

